# Altered dynamic functional connectivity in the primary visual cortex in patients with primary angle-closure glaucoma

**DOI:** 10.3389/fnins.2023.1131247

**Published:** 2023-02-02

**Authors:** Dong-Jin Li, Bing-Lin Huang, Yuan Peng, Ling-Yan Liang, Hui Liu

**Affiliations:** ^1^Health Management Center, The First People’s Hospital of Jiujiang City, Jiujiang, Jiangxi, China; ^2^College of Clinical Medicine, Jiangxi University of Traditional Chinese Medicine, Nanchang, Jiangxi, China; ^3^Department of Ophthalmology, Affiliated Hospital of Jiangxi University of Traditional Chinese Medicine, Nanchang, Jiangxi, China; ^4^College of Graduate, Jiangxi University of Traditional Chinese Medicine, Nanchang, Jiangxi, China; ^5^Department of Ophthalmology, Jiangxi Provincial People’s Hospital, Nanchang, Jiangxi, China

**Keywords:** dynamic functional connectivity, functional magnetic resonance imaging, primary angle-closure glaucoma, primary visual cortex, machine learning

## Abstract

**Purpose:**

Glaucoma is the main blindness-causing disease in the world. Previous neuroimaging studies demonstrated that glaucoma not only causes the loss of optic ganglion cells but also leads to the abnormal function of the optic nerve pathway and the visual cortex. However, previous studies also reported that patients with glaucoma have dysfunction in the visual cortex in a static state. Whether or not patients with primary angle-closure glaucoma (PACG) were accompanied by dynamic functional connectivity (FC) changes in the primary visual cortex (V1) remains unknown.

**Methods:**

A total of 34 patients with PACG (23 men and 11 women) and 34 well-matched healthy controls (HCs) were enrolled in the study. The dynamic functional connectivity (dFC) with the sliding window method was applied to investigate the dynamic functional connectivity changes in the V1.

**Results:**

Compared with HCs, patients with PACG showed increased dFC values between left V1 and bilateral calcarine (CAL). Meanwhile, patients with PACG showed increased dFC values between right V1 and bilateral CAL.

**Conclusion:**

Our study demonstrated that patients with PACG showed increased dFC within the visual network, which might indicate the increased variability FC in the V1 in patients with PACG.

## Introduction

Primary angle-closure glaucoma (PACG) is a serious irreversible blinding eye disease. PACG leads to increased intraocular pressure, which in turn leads to the apoptosis of optic ganglion cells. PACG is characterized by ocular pain and visual field defects, which might lead to blindness in advanced cases. PACG is more common in Asians than Europeans and Africans, with >80% of PACG cases worldwide occurring in Asia. At present, the prevalence of PACG is 0.75% in adult Asians worldwide (Cheng et al., 2014). Glaucoma not only leads to the loss of retinal ganglion cells but also leads to abnormal changes in the optic nerve pathway and visual center function. Retinal ganglion cell death could leads to anterograde and retrograde in visual pathway (Lawlor et al., 2018).

With the development of functional magnetic resonance imaging (fMRI) technology, multimodal MRI techniques, including diffusion tensor imaging (DTI) and fMRI, have been used widely to investigate the functional changes of the visual cortex in glaucoma. Numerous studies have shown that patients with glaucoma have primary visual cortex (V1) and V1-related dysfunction. Li et al. (2017) demonstrated that patients with PACG have decreased functional connectivity (FC) between the left V1 and right V2 and increased FC between the left V1 and higher cognitive cortex.

Meanwhile, Jiang et al. (2020) reported that patients with PACG showed abnormal effective connectivity between V1 and the higher visual area, motor cortices, somatosensory cortices, and frontal lobe. Graham et al. (2021) found that dogs with unilateral PACG showed reduced fractional anisotropy (FA) in the visual pathway. Nuzzi et al. (2018) demonstrated that patients with glaucoma showed abnormal FC changes in the visual pathway. In addition, Pankowska et al. (2022) reported that the patients with advanced glaucoma showed an increase in the gray matter thickness in the V1 region. Meanwhile, early glaucoma was associated with reduced thickness in the right lateral occipital gyrus and the left lingual gyrus (Pankowska et al., 2022). Haykal et al. (2022) reported that patients with primary open-angle glaucoma (POAG) showed a loss of both axonal coherence and density in the pregeniculate visual pathways, while the postgeniculate pathways exhibited a loss of axonal coherence. Meanwhile, patients with POAG showed a decrease in fiber density and fiber-bundle cross-section in the pregeniculate optic tracts, whereas the postgeniculate optic radiation showed a decrease in fiber density (Haykal et al., 2019).

Thus, combined with our findings, previous studies demonstrated that patients with glaucoma were along with visual pathway and visual cortex dysfunction. However, the abovementioned studies focused mainly on static brain activity changes in the visual cortex. It is recognized increasingly that the functional activity changes in the brain not only are constant over time but also show dynamic changes over time.

The human brain is a complex dynamic system capable of non-stationary neural activity. The human brain showed inherently dynamic activity, which is related to the functional ability of neural networks. The dynamics of neural activity in the brain is closely related to neurophysiological activity. Growing evidence demonstrated that the fluctuations in FC are of neural origin and are also even more prominent during the resting period when mental activity is unconstrained. Temporal variability of the dynamic brain activity is related closely to various neurophysiological activities, including working memory (Shunkai et al., 2021), vision (Di and Biswal, 2020), and cognition (Patil et al., 2021). Therefore, recent studies have shown that the dynamics of brain activity can better reflect neural activity. Dynamic FC with the sliding window method is applied widely to investigate the dynamic neural activity changes in neuroimaging studies (Yuan et al., 2019; Li et al., 2020; Tsurugizawa and Yoshimaru, 2021). Previous neuroimaging studies demonstrated that patients with PACG showed abnormal neural activity changes in the visual cortex and visual-related cortex. However, there have been no studies on the dynamic FC changes in the V1 in patients with PACG. Thus, we hypothesized that patients with PACG might be associated with dynamic FC changes in the V1.

Based on these assumptions, our study is the first to determine whether or not patients with PACG are associated with the dynamic FC changes in the V1. Moreover, we chose the dFC maps as a feature of machine-learning classification. Machine learning for MRI-related neuroimaging has been widely applied in the diagnosis of nervous system diseases. In addition, the support vector machine (SVM) method is the most commonly used supervised machine learning algorithm for MRI classification related to random forests, decision trees, and convolutional neural networks.

## Materials and methods

### Participants

In total, 34 patients with PACG and 34 healthy controls (matched for sex and age) were recruited in this study. The inclusion criteria for individuals with PACG were as follows: (1) the intraocular pressure is higher than 21 mmHg; (2) without other eye diseases (cataract, optic neuritis, high myopia, etc.); (3) no medical treatment of PACG; (4) eliminate patients with primary open-angle glaucoma (POAG) and normal tension glaucoma (NTG); and (5) without brain lesions including cerebral hemorrhage and cerebral infarction.

The inclusion criteria for healthy controls were as follows: (1) normal visual acuity [>1.0] in both eyes; (2) no ophthalmic diseases (i.e., optic neuritis, cataract, keratitis, etc.). This study was performed in accordance with the tenets of the Declaration of Helsinki. Each participant provided written informed consent before inclusion in the study.

### MRI acquisition and experimental procedure

Magnetic resonance imaging scanning was performed on a 3-tesla magnetic resonance scanner (Discovery MR 750W system; GE Healthcare, Milwaukee, WI, USA) with the eight-channel head coil. Functional images were obtained using a gradient-echo-planar imaging sequence. fMRI scanning parameter:repetition time = 2,000 ms, echo time = 25 ms, thickness = 3.0 mm, gap = 1.2 mm, acquisition matrix = 64 × 64, field of view = 240 mm^2^ × 240 mm^2^, flip angle = 90°, voxel size = 3.6 mm^3^ × 3.6 mm^3^ × 3.6 mm^3^, and 35 axial slices. Before fMRI scanning, all study subjects were informed of all the caveats of the experiment and were asked to remove any metal objects. Then, during the fMRI scanning, all subjects were asked to keep their eyes closed and relaxed without falling asleep.

### fMRI data analysis

All preprocessing was performed using the toolbox for Data Processing and Analysis of Brain Imaging (DPABI)^1^ (Yan et al., 2016), which is based on the Statistical Parametric Mapping (SPM12)^2^ implemented in MATLAB 2013a (MathWorks, Natick, MA, USA) and the details of steps according to a previous study (Chen et al., 2022).

### dFC analysis

The dFC method was performed using DPABI software. Based on previous studies (Wen et al., 2018; Qi et al., 2021; Tong et al., 2021), left V1(*x* = –8, *y* = –76, *z* = 10) and right V1 (*x* = 7, *y* = –76, *z* = 10) with 6 mm radius. Specifically, the R-fMRI indices mentioned above were computed with hamming windows (window length = 30 TR, window step = 1 TR). To avoid the introduction of spurious fluctuations during the sliding window select, the minimum window length should be larger than 1/*f*min, where *f*min is the minimum frequency of the time series.

### Support vector machine (SVM) analysis

The support vector machine (SVM) algorithm for binary classification is implemented on the Pattern Recognition for Neuroimaging Toolbox (PRoNTo) software^3^ (Schrouff et al., 2013). The details of the steps for the SVM method were as follows: (1) The dFC maps were selected as a classification feature. (2) The leave-one-out cross-validation (LOOCV) was applied to perform the SVM classifier validation. (3) The permutation test was applied to assess the statistical significance of the total accuracy of this classification. (4) The total accuracy, specificity, sensitivity, and area under the receiver operating characteristic curve (AUC) were determined to assess and classify the patients with PACG and the HCs.

### Statistical analysis

The independent sample *t*-test was used to investigate the clinical features between the two groups. In this study, a one-sample *t*-test was applied to assess the group the mean of dFC maps within two groups, and the two-sample *t*-test was used to compare the two group differences in the dFC maps between the two groups using the Gaussian random field (GRF) method (two-tailed, voxel-level *p* < 0.01, GRF correction, cluster-level *p* < 0.05).

## Results

### Analysis of ophthalmic clinical data

There were no statistically significant differences between the PACG and HC groups in gender or age. However, there were significant differences between the two groups in visual acuity. More details of the results are shown in Table 1.

**TABLE 1:** Clinical characteristics for health controls (HCs) and patients with primary angle-closure glaucoma (PACG).

	PACG group	HC group	*T*-Value	*P*-Value
Sex (male/female)	23/11	23/11	N/A	0.780
Age (years)	45.15 ± 14.95	45.30 ± 13.87	−0.038	0.970
BCVA-OD	0.44 ± 0.27	1.16 ± 0.16	−11.474	<0.001*
BCVA-OS	0.43 ± 0.37	1.19 ± 0.16	−9.352	<0.001*

Chi-square test for sex. Independent *t*-test was used for other normally distributed continuous data.

Data are presented as mean ± standard deviation.

HC, healthy control; BCVA, best-corrected visual acuity; OD, oculus dexter; OS, oculus sinister; N/A, not applicable; R, right.

*p < 0.001.

### Different dFC values between two groups

**FIGURE 1:**
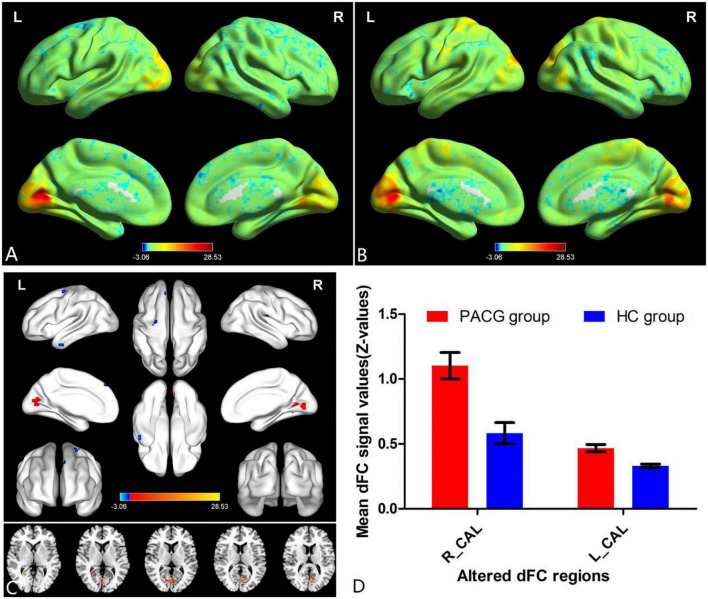
One-sample *t*-test results of dynamic functional connectivity (dFC) in left V1 maps within primary angle-closure glaucoma (PACG) group **(A)** and HC group **(B)**; significant dFC differences in left V1 between two groups **(C)**; the mean of altered dFC values of left V1 between the patients with PACG and HCs **(D)**. PACG, primary angle-closure glaucoma; HC, health control; V1, primary visual cortex; dFC, dynamic functional connectivity; CAL, calcarine; R, right; L, left.

**FIGURE 2:**
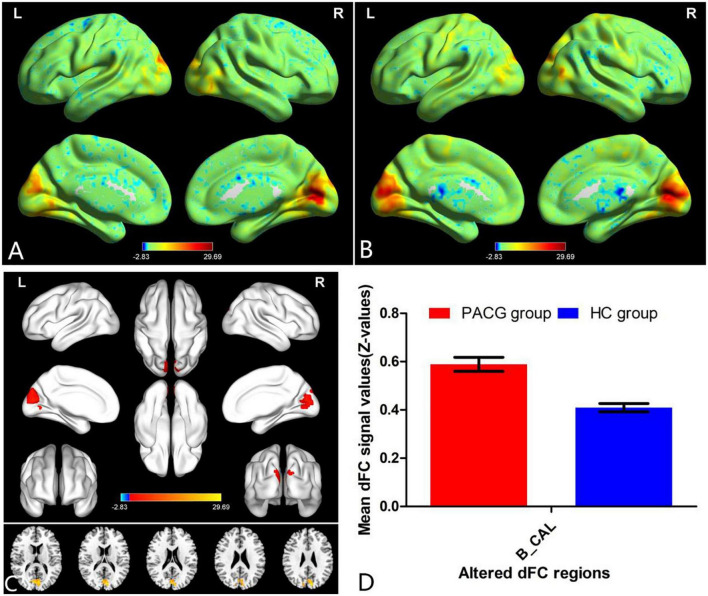
One-sample *t*-test results of dFC in right V1 maps within PACG group **(A)** and HC group **(B)**; significant dFC differences in right V1 between two groups **(C)**; the mean of altered dFC values of right V1 between the patients with PACG and HCs **(D)**. PACG, primary angle-closure glaucoma; HC, health control; V1, primary visual cortex; dFC, dynamic functional connectivity; CAL, calcarine; B, bilateral; R, right; L, left.

The group means of dFC maps of the PACG and HC are shown in Figures 1A, B, 2A, B. Compared with HCs, patients with PACG showed increased dFC values between left V1 and bilateral calcarine (CAL) (Figure 1C and Table 2). The mean values of altered dFC in left V1 values were shown with a histogram (Figure 1D). Meanwhile, patients with PACG showed increased dFC values between right V1 and bilateral calcarine (CAL) (Figure 2C and Table 3). The mean values of dFC in right V1 values were shown with a histogram (Figure 2D).

**TABLE 2:** Different dynamic functional connectivity (dFC) values in the left V1 between two groups.

Condition	Brain regions	BA	Peak T-scores	MNI coordinates (x, y, z)	Cluster size (voxels)
PACG > HC	Calcarine_R	–	3.5784	24, −57, 3	10
PACG > HC	Calcarine_L	–	4.0202	−6, −69, 12	51

Different dFC values in left V1 between two groups (voxel-level *p* < 0.01, GRF correction, cluster-level *p* < 0.05).

PACG, primary angle-closure glaucoma; HC, health control; MNI, Montreal Neurological Institute; dFC, dynamic functional connectivity; R, right; L, left.

**TABLE 3:** Different dFC values in the right V1 between two groups.

Condition	Brain regions	BA	Peak T-scores	MNI coordinates (x, y, z)	Cluster size (voxels)
PACG > HC	Calcarine_B	–	4.5846	21, −75, 3	237

Different dFC values in right V1 between two groups (voxel-level *p* < 0.01, GRF correction, cluster-level *p* < 0.05).

PACG, primary angle-closure glaucoma; HC, health control; MNI, Montreal Neurological Institute; dFC, dynamic functional connectivity; B, bilateral.

### Support vector machine (SVM) result

**FIGURE 3:**
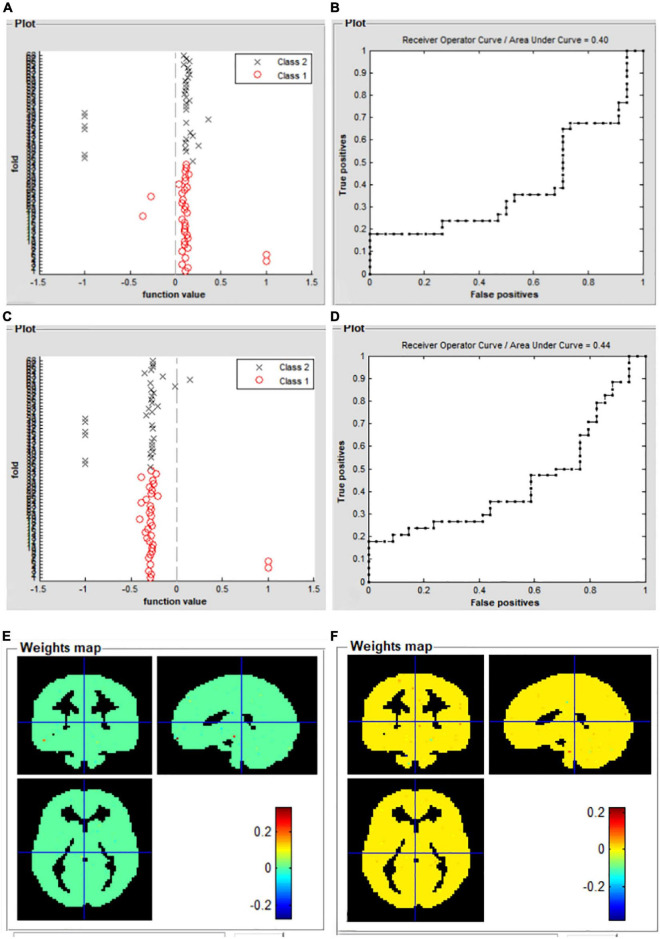
Support vector machine classification results based on the mean dFC maps. Function dFC in left V1 value of SVM algorithm for two groups (class 1: PACG group; class 2: HC group) **(A)**; the receive operating characteristic curve of the SVM classifier **(B)**; function dFC in left V1 value of SVM algorithm for two groups (class 1: PACG group; class 2: HC group) **(C)**; the receive operating characteristic curve of the SVM classifier **(D)**; weight maps for SVM models for left V1 **(E)**; weight maps for SVM models for right V1 **(F)**. PACG, primary angle-closure glaucoma; HC, health control; V1, primary visual cortex; dFC, dynamic functional connectivity; SVM, support vector machine.

For the left V1 map, the total classification accuracy was 55.88%. Function value of SVM algorithm for two groups (in scatter diagram. class 1: PACG group; class 2: HC group) (Figure 3A). A receive operating characteristic curve of the binary classifiers was generated to evaluate the system’s performance in distinguishing individuals with PACG from HCs and the AUC was 0.40 (Figure 3B).

For the right V1 map, total classification accuracy was 51.47%. Function value of SVM algorithm for two groups (in scatter diagram. class 1: PACG group; class 2: HC group) (Figure 3C). A receive operating characteristic curve of the binary classifiers was generated to evaluate the system’s performance in distinguishing individuals with PACG from HCs and the AUC was 0.44 (Figure 3D). Weight maps for SVM models for left V1 (Figure 3E), and weight maps for SVM models for right V1 (Figure 3F).

## Discussion

Our study is the first to investigate the dFC alterations of the V1 in patients with PACG. Patients with PACG showed increased dFC values between the left V1 and bilateral CAL. Meanwhile, patients with PACG showed increased dFC values between the right V1 and bilateral CAL related to the HC group.

Our most interesting finding is that the patients with PACG showed an increased dFC within the visual network. As we all know, the most important pathological mechanism of glaucoma is the loss of retinal ganglion cells. Meanwhile, retinal ganglion cell death can lead to anterograde and retrograde retinal ganglion cell degeneration (Lawlor et al., 2018). Thus, the damage to the optic nerve leads to abnormal visual signaling in the visual cortex. Thus, we speculated that the reduced afferent visual signals may lead to increased variability and decreased stability of primary visual cortex neural activity. Hernowo et al. (2011) reported that reduced volume in the visual pathway was found in patients with glaucoma. Chen et al. (2013) found that patients with glaucoma showed decreased gray matter (GM) volume in the visual cortex. Frezzotti et al. (2014) also demonstrated that patients with POAG had brain atrophy in both the visual cortex and other distant GM regions (the frontoparietal cortex, the hippocampi, and the cerebellar cortex). Wang et al. (2016) found that patients with glaucoma had significantly reduced cortical thickness in the right frontal pole and decreased GM volume in LGN, the right V1, and the left amygdala. Thus, brain structural atrophy in VI may lead to increased dFC values of V1 in patients with glaucoma. We speculated that the abnormal brain structure may lead to local neural activity flexibility changes. Consistent with these findings, our study found that patients with PACG showed increased dFC values within the visual network. Increased dFC might reflect increased neural activity flexibility in the visual cortex, which might indicate visual function compensation in patients with PACG.

In addition, in our study, the SVM method was used to investigate the predictive values of classifying individual patient populations with PACG versus HCs. For the left V1 map, the total classification accuracy was 55.88%. A receiver operating characteristic (ROC) curve of the binary classifiers was generated to evaluate the system’s performance in distinguishing individuals with PACG from HCs and the area under the ROC curve (AUC) was 0.40. For the right V1 map, the total classification accuracy was 51.47%. A ROC curve of the binary classifiers was generated to evaluate the system’s performance in distinguishing individuals with PACG from HCs and the AUC was 0.44. The SVM algorithm was trained with input data labeled previously by PACG or HCs to predict the desired outcome. This powerful multivariate analysis enabled us to make clinical predictions at the individual subject level.

However, there were some limitations to the study. First, the sample size of our study was small. Second, blood oxygen level-dependent (BOLD) signals would be influenced by a variety of physiological noises such as breathing and heartbeat. Third, the machine noise of MRI can also have an effect on the BOLD signal. Finally, a different time window length might affect the results. We would select a different window length in the future study for the stability of the results.

In conclusion, our study demonstrated that patients with PACG showed an increased dFC within the visual network, which might be indicative of an increased variability of FC in the primary visual cortex. Our study also provides an important imaging reference to aid the understanding of the mechanism of nerve damage in the visual center of patients with glaucoma.

## Data availability statement

The raw data supporting the conclusions of this article will be made available by the authors, without undue reservation.

## Ethics statement

The studies involving human participants were reviewed and approved by tenets of the Declaration of Helsinki of Jiangxi Provincial People’s Hospital. The patients/participants provided their written informed consent to participate in this study. Written informed consent was obtained from the individual(s) for the publication of any potentially identifiable images or data included in this article.

## Author contributions

D-JL, B-LH, YP, L-YL, and HL contributed to the data collection, statistical analyses, and wrote the manuscript. B-LH, YP, L-YL, and HL designed the protocol and contributed to the MRI analysis and designed the study, oversaw all clinical aspects of study conduct, and manuscript preparation. All authors contributed to the article and approved the submitted version.

## Footnotes

1
http://www.rfmri.org/dpabi

2
http://www.fil.ion.ucl.ac.uk

3
http://www.mlnl.cs.ucl.ac.uk/pronto/prtsoftware.html
